# Effect of alkylresorcinols on the formation of Nε‐(carboxymethyl)lysine and sensory profile of wheat bread

**DOI:** 10.1002/fsn3.2018

**Published:** 2020-11-18

**Authors:** Jie Liu, Zihui Yang, Yiming Hao, Ziyuan Wang, Lin Han, Meng Li, Ning Zhang, Haitao Chen, Yingli Liu, Hongyan Li, Jing Wang

**Affiliations:** ^1^ China‐Canada Joint Lab of Food Nutrition and Health (Beijing) Beijing Technology & Business University (BTBU) Beijing China; ^2^ Beijing Advanced Innovation Center for Food Nutrition and Human Health Beijing Technology & Business University Beijing China

**Keywords:** alkylresorcinols, Nε‐(carboxymethyl) lysine, odor activity value, sensory profile, volatile compounds, wheat bread

## Abstract

Alkylresorcinols (ARs) are important bioactive components in wheat bran which have been used as biomarkers for whole grain wheat consumption. In this study, the impact of ARs on the formation of Nε‐(carboxymethyl)lysine (CML), the main component of dietary advanced glycation end products which could induce chronic disease was analyzed. Moreover, the influence of the addition of ARs on the sensory profiles of wheat bread was evaluated. ARs supplementation (0.03%, 0.1%, and 0.3% w/w) could significantly decrease the formation of CML by 21.70%, 35.11%, and 42.18%, respectively, compared with the control. Moreover, ARs‐supplemented bread achieved a higher score in overall acceptability and buttery‐like aroma through sensory evaluation. The volatile compounds in bread supplemented with ARs were characterized by headspace solid phase microextraction‐gas chromatography‐mass spectrometry (HS‐SPME‐GC‐MS), among which acetoin, 2,3‐butanedione, 3‐methyl‐1‐butanol, 2‐phenylethanol, and 2‐methylbutanal were confirmed as the main volatile compounds through determination of odor activity value. In addition, ARs supplementation had no negative impact on the chewiness, hardness, and springiness of bread. These findings demonstrated that ARs could be applied as potential food additives to improve the quality and sensory profile of bread.

## Graphical abstract

The addition of ARs could significantly decrease the formation of CML in bread. ARs supplementation improved the quality and sensory profiles of wheat bread. ARs addition showed no negative impact on the texture characteristics of bread.

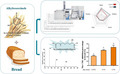

## 1 INTRODUCTION

During thermal processing, Maillard reaction not only provides food with distinctive flavor and browned color, but also results in potentially harmful substances, such as acrylamide, heterocyclic amines, and advanced sglycation end products (AGEs) (Teng et al., 2018). AGEs are a diverse group of lysine (Lys)‐ or arginine (Arg)‐derived products, which are formed at the intermediate stage of Maillard reaction via the amadori route, dicarbonyl route, or radical reaction route (Zhao et al., 2019). As the main component of AGEs, Nε‐(carboxymethyl) lysine (CML) is also the most prevalent detection marker for AGEs in food analysis (Ou et al., 2017). The accumulation of CML could induce chronic diseases such as atherosclerosis, nephropathy, retinopathy, and diabetes (Li et al., 2015). As the intake of AGEs by human body was largely derived from food, it was of vital importance to avoid the formation of CML during food processing.

At present, studies on CML inhibition were mainly conducted in chemical or biological model other than real thermally processed food, thus the effects of CML inhibitors (e.g., phenolic compounds) on food flavor and texture were not fully investigated. Phenolic compounds such as gallic acid, (+)‐catechin, caffeic acid, and ferulic acid exhibited obviously inhibitory activity on the formation of CML in bread, however they could cause the reduction of pleasant flavors (Mildner‐Szkudlarz et al., 2017). Therefore, the selection criteria of CML inhibitors should be given a comprehensive evaluation of the effects on food safety, flavor, and texture properties which would be valuable for food processing and development.

Alkylresorcinols (ARs) are important bioactive components in wheat bran, which can be used as biomarkers for whole grain wheat consumption (Liu et al., 2020). In recent years, ARs are found to possess multiple potential health benefits, such as anti‐oxidant (Wang et al., 2019), anti‐inflammatory (Liu et al., 2018), and anti‐obesity properties (Oishi et al., 2015). However, the effect of ARs on the formation of CML, and its impact on the flavor profile in a real food system have not been reported so far. In the present study, the impact of ARs on the formation of CML in wheat bread and its addition on the sensory property including flavor and texture profiles of bread were explored. The results provide an alternative for using ARs as functional ingredients to reduce dietary content of CML and improve bread quality and flavor.

## 2 MATERIALS AND METHODS

### 2.1 Materials

Wheat alkylresorcinols were prepared according to the method reported in our previous study (Liu et al., 2018). Wheat flour (10.5% protein, 1.7% fat, and 75.5% carbohydrates) were purchased from Beijng Er Shang Group (Beijing, China). Baker's yeast was obtained from Angel Yeast (Hubei, China). 2‐Methyl‐3‐heptanone were obtained from Sigma Aldrich (St. Louis, Mo, USA). Nε‐(carboxymethyl)lysine was purchased from J&K Scientific Ltd. (Beijing, China).

### 2.2 Bread preparation

Wheat breads were prepared using 100 g of wheat flour, 75 g of water, 1.5 g of dry yeast, 1.6 g of salt, 6.3 g of sugar, and 2 g of corn oil. ARs were ground into powder and mixed with wheat flour at different levels (0%, 0.03%, 0.1%, and 0.3% w/w). After being completely mixed, kneaded, and rested for 20 min, the dough was fermented for 1h and baked for 15 min at 170℃ using a bread maker (NB‐H3202, Panasonic, Japan). Subsequently, the baked bread was cooled to room temperature and divided into evenly sized pieces without separating the crust and crumb for later analysis.

### 2.3 LC–MS analysis

The method used to determine CML was according to a previous study with minor modification (Tareke et al., 2013). Briefly, 0.1 g of bread sample was hydrolyzed by 1 ml of 6 M HCl at 110°C for 24 hr (Jiao et al., 2017) and then dried by nitrogen purge. Subsequently, dried hydrolysate was dissolved in 1 ml of water and filtered through a 0.22 μm filter before analysis using Agilent Technology 6420 Triple Quad LC‐MS (Milford, MA, USA) with a Shimadzu Inertsil ODS‐SP C18 column (4.6 mm × 250 mm id, 5 μm). The injection volume was 10 μL with a flow rate of 0.2 ml/min, and the oven temperature was set at 35°C. The mobile phase consisted of 0.1% FA ‐water as solvent A and acetonitrile as solvent B. The gradient elution program was started with 10% of solvent B at the beginning, after 1 min, increasing with a linear gradient to 40% B in 3.5 min, then held 40% B until 21mins. After that, the gradient program was returned to the initial conditions. The MRM‐MS was conducted at a positive ionization mode and the optimized parameters were as following: spray voltage of 4 kV, source temperature at 350°C. For CML detection, the transitions m/z 205.0 → 84.0 (135 V, 30eV) were used for quantification.

### 2.4 Volatile compounds analysis

The volatile compounds in bread were extracted by headspace solid phase microextraction (HS‐SPME) and analyzed using GC‐MS (Trace‐1300 ISQ‐Mass, Thermo Fisher Scientific, San Jose, CA, USA) according to a previous report with some modification (Pu et al., 2019). Briefly, 2 g bread sample was mixed with 0.5 μL 2‐methyl‐3‐heptanone (0.816 μg/μl in *n*‐hexane) loaded in a 20 ml glass vial. After equilibration, the mixture was incubated in a water bath for 30 min at 80°C. Volatile compounds were adsorbed using SPME needle with a 65 μm PDMS/DVB fiber for 20 min in the headspace, followed by desorption at 250°C for 5 min in GC‐MS with a TG‐WAXMS Column (30 m x 0.25 mm x 0.25 µm; Thermo Fisher Scientific Inc. Waltham, MA, USA). The program of column temperature was set as follows: 40°C, held for 1 min; increased by 2°C/min to 100°C, held for 1 min; increased by 4.5°C/min to 170°C, held for 1 min; increased by 3°C/min to 220°C, held for 1 min. The carrier gas was Helium with a flow rate of 1.2 ml/min. The temperature of ion source and injection port was 250°C. The split ratio was set to splitless. The ionization mode was EI, and the electron energy was 70 eV. The mass scan range was 40–400 *m/z*.

### 2.5 Identification and quantitative analysis of volatile compounds

Identification of volatile compounds was carried out by comparing their MS data and calculation of the retention indices (RIs) with the database according to NIST (MS Search 2.0). Semi‐quantification of the volatile compounds was conducted using 2‐methyl‐3‐heptanone as the internal standard (IS). The concentration of the target compounds based on their peak area was calculated by comparing the concentration of IS and the peak area of IS. All of the compounds were quantified as 2‐methyl‐3‐heptanone equivalents.

### 2.6 Odor activity value (OAV)

The contribution of the volatile compound to the overall aroma of the bread was evaluated by their odor activity value (OAV). OAV was calculated as the ratio of the individual compound concentration to their odor thresholds (Caliari et al., 2015).

### 2.7 Sensory evaluation

The sensory analysis was carried out as described by Souza‐Borges and Conti‐Silva (2018). The bread samples were cooled for three hours to room temperature before serving to an untrained sensory panel (7 males, 8 females) composing of 15 consumers. The consumers received a sample of each bread on a dish encoded with random 3‐digits. Everyone should score for five sensorial parameters, including appearance, flavor, hardness, springiness, and overall acceptability. Sensory evaluation of breads was carried out using ten‐point hedonic scale (1–2: dislike extremely; 3–4: dislike; 5–6: neither like nor dislike; 7–8: like; 9–10: like extremely).

A quantitative descriptive analysis was applied for evaluating bread samples. Before the quantitative descriptive analysis, a common bread was divided into evenly sized pieces and served to nine trained panelists to discuss aroma compositions of the sample, until everyone agreed to use them as the attributes. Panelists scored the intensities of the buttery, flowery, malty, sweet, and fatty odor of breads based on a 0 to 10 scale (0: not detectable; 1–2: extremely weak intensity; 3–4: weak intensity; 5–6: medium intensity; 7–8: high intensity; 9–10: extremely high intensity). The sensory scores were calculated as the average score of each attribute plus the standard deviation.

### 2.8 Texture profile analysis

CT3 texture analyzer (Brookfield, Massachusetts, USA) with TA4/1000 cylindrical probe was used for texture profile analysis (TPA). The instrument parameters were set according to American Association of Cereal Chemists (AACC) standard (74–09). The bread samples were cooled for 30 min at room temperature and sliced into pieces of 25 mm thickness before TPA analysis. The determinations were calculated in triplicate for each sample.

### 2.9 Statistical analysis

All results were represented as mean ± standard deviation (*SD*). Statistical significance was determined using Student's *t* test in instances of two experimental groups. For more than two groups, statistical evaluation of the results was performed by one‐way ANOVA with Duncan's test. *P*‐values of less than 0.05 were considered statistically significant.

## 3 RESULTS AND DISCUSSION

### 3.1 Effects of ARs on CML formation in wheat bread

**Figure 1:**
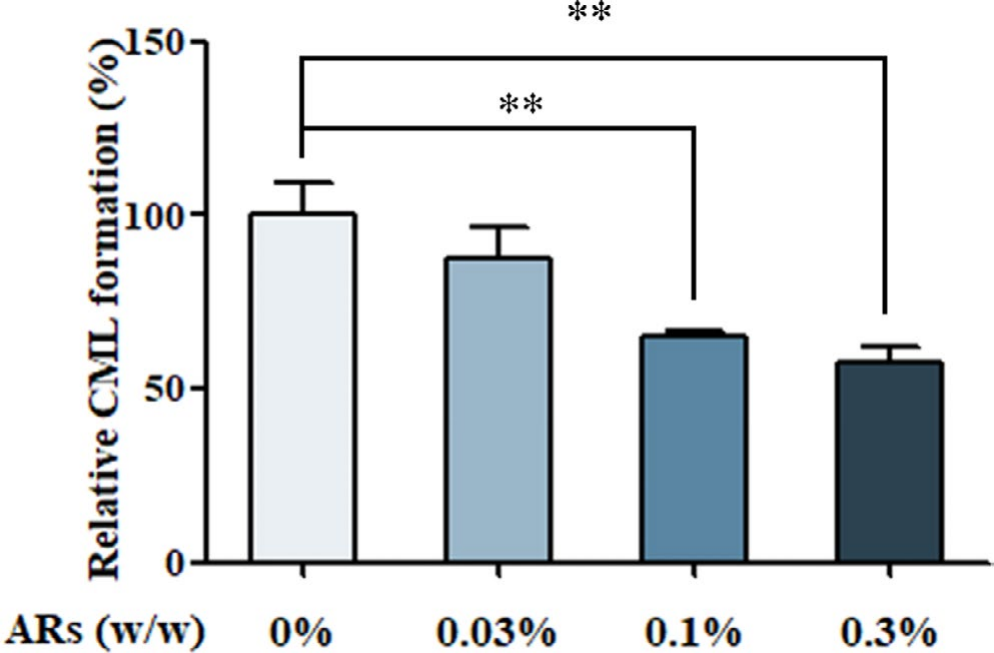
The effect of different concentrations of ARs (0.03%, 0.1%, and 0.3% w/w) on the formation of CML in bread. Statistical significance was determined by one‐way ANOVA with Duncan's test for multiple‐group comparisons. Level of significance: * and ** indicates significance at *p* < .05 and *p* < .01, respectively

ARs‐supplemented bread (ARs‐B) at the dose of 0.03%, 0.1%, and 0.3% (w/w) could significantly decrease the formation of CML by 21.70%, 35.11%, and 42.18%, respectively, compared to the bread without addition (Figure 1). The inhibitory efficiency of ARs on the formation of CML in wheat bread sample might be ascribed to its chemical structure, the molecule backbone of which is resorcinol (1,3‐dihydroxybenzene). It has been reported that flavonoids owning phenolic hydroxyl groups could bound reactive dicarbonyl species and hinder the generation of CML (Mildner‐Szkudlarz et al., 2017; Yoon & Shim, 2015). Another report indicated that flavonoids and polyphenols could inhibit the formation of CML in food systems by inhibiting the oxidation of glucose and scavenging free radicals (Rice‐Evans et al., 1996). Our previous study also indicated that ARs could significantly inhibit oxidative damage and reactive oxygen species (ROS) generation by improving antioxidant enzymes activities, such as catalase, NAD(P)H: quinone oxidoreductase 1 (NQO1) and heme‐oxygenase‐1 (Wang et al., 2019). Therefore, the inhibitory ability of ARs on CML formation may due to the elimination of free radicals, which were important intermediates of CML formation.

### 3.2 Sensory characterization and acceptability of ARs‐supplemented bread

**Figure 2:**
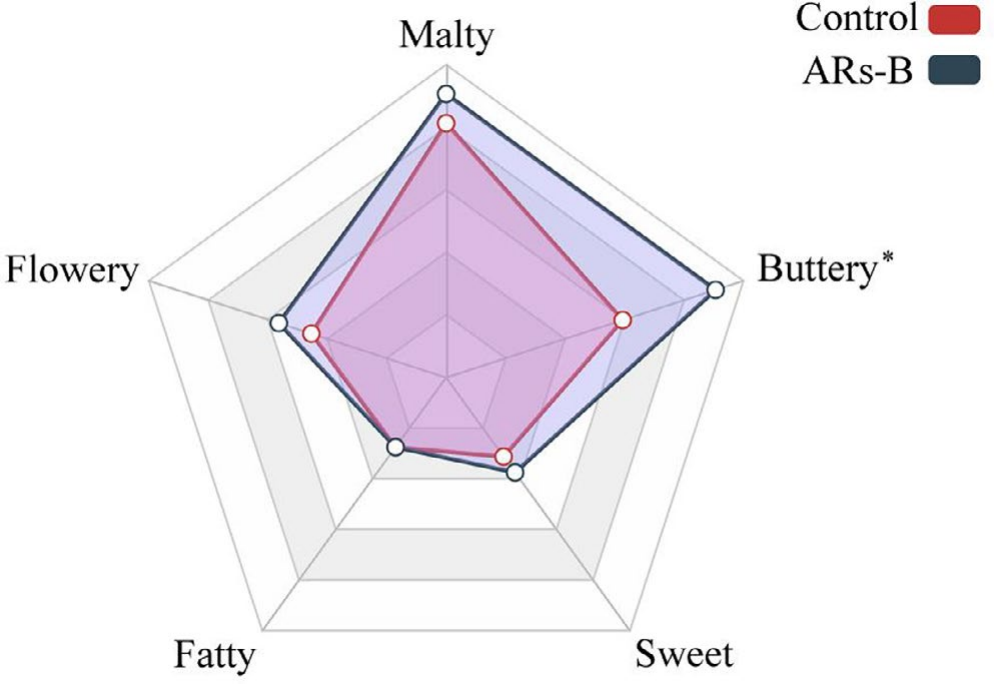
Sensory profiles of control and ARs‐B. Significant differences compared with control bread group are assessed by Student's *t* test. Level of significance: * indicates significance at *p* < .05. Control: Normal bread; ARs‐B: Bread supplemented with ARs (0.3% w/w)

**Figure 3:**
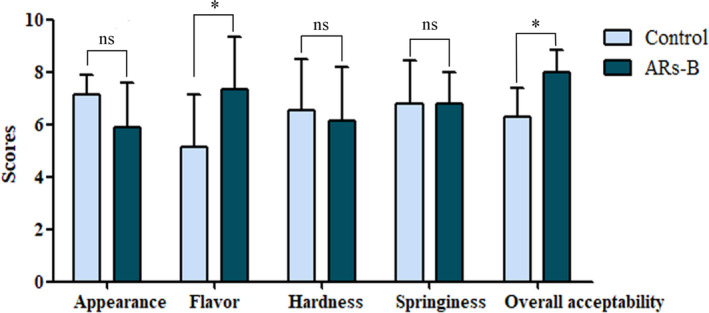
The sensory acceptability scores of bread samples obtained from control and ARs‐B samples. Significant differences compared with control bread group are assessed by Student's *t* test. Level of significance: * indicates significance at *p* < .05. Control: Normal bread; ARs‐B: Bread supplemented with ARs (0.3% w/w)

Bread with or without ARs supplementation was subjected to aroma profile analysis, which was performed by the evaluation of the organoleptic qualities of bread using five aromas descriptors including “buttery”, “flowery”, “malty”, “sweet”, and “fatty” (Majcher et al., 2019; Markus et al., 2017; Zhu et al., 2015). Based on the sensory evaluation scores, ARs‐B exhibited predominantly buttery‐like aroma characteristics compared with the control (Figure 2). Moreover, the results showed there were equal degrees of acceptability in relation to appearance, hardness, and springiness. However, ARs‐B had a higher score in flavor and overall acceptability compared with control bread, suggesting ARs addition had a positive effect on the quality and flavor of wheat bread in some extent (Figure 3). Especially in Table 1, the results of correlation analysis obtained from control and ARs‐B which illustrated that ARs supplement resulted in a significant positive correlation between flavor and overall acceptability. The results indicated that ARs may improve the overall acceptability of wheat bread by promoting the flavor characteristics of bread.

**Table 1:** The correlation of sensory acceptability scores obtained from control and ARs‐B samples

Sensory acceptability scores			Pearson correlation
Hardness	versus	springiness	0.563*
Hardness	versus	appearance	−0.045
Hardness	versus	flavor	−0.025
Hardness	versus	overall acceptability	−0.113
Springiness	versus	appearance	0.178
Springiness	versus	flavor	0.426
Springiness	versus	overall acceptability	0.365
Appearance	versus	flavor	−0.146
Appearance	versus	overall acceptability	−0.127
Flavor	versus	overall acceptability	0.887**

^a^ Control: Normal bread.

^b^ ARs‐B: Bread supplemented with ARs (0.3% w/w).

* Significance at *p* < .05

** Significance at *p* < .01.

### 3.3 Effect of ARs on volatile profile of wheat bread

Total of 25 volatile compounds were detected from wheat ARs extraction (Table 2). And a total of 33 volatile compounds were detected from ARs‐supplemented bread, including 9 alcohols, 3 esters, 6 acids, 8 aldehydes, 2 ketones, and 5 heterocycles (Table 3). A Student's *t* test was applied to investigate the effect of ARs addition on the volatile profile of wheat bread. Significant differences were found between control and ARs‐B in different volatile families including alcohols, represented by 3‐methyl‐1‐butanol, 1‐hexanol, and 2‐phenylethanol; ketones, represented by acetoin and 2,3‐butanedione and heterocycles, represented by maltol (Table 3).

**Table 2:** Composition of volatile compounds in ARs

NO		Volatile	RT	RI^a^/RI^b^	Odor descriptor
1	Alcohols	3‐Methyl‐1‐butanol	10.29	1143/1185	Whiskey, malt, burnt
2		1‐Pentanol	12.38	1184/1232	Balsamic
3		1‐Hexanol	18.01	1287/1336	Green,resin, flower
4		1‐Octen‐3‐ol	23.89	1381/1399	Mushroom
5		2‐Ethylhexanol	26.25	1425/1475	Green,rose
6		1‐Octanol	30.33	1488/1534	Chemical, metal, burnt
7		Benzyl alcohol	42.92	1793/1837	Sweet, flower
8		2‐Phenylethanol	43.89	1830/1875	Flowery, yeast‐like, honey
9		1‐Dodecanol	45.96	1912/1953	Fat, wax
10	Esters	Ethyl lactate	17.13	1270/1316	Fruit
11		Ethyl 3‐hydroxybutyrate	27.3	1438/1483	Marshmallow
12		Methyl salicylate	39.67	1688/1735	Peppermint
13	Acids	2‐Methylbutanoic acid	35.87	1590/1620	sweat,cheese
14		Pentanoic acid	38.61	1672/1712	Sweat
15		Hexanoic acid	42.11	1766/1802	Sweat
16		Octanoic acid	47.89	1993/2038	Sweat, cheese
17		Nonanoic acid	50.75	2103/2144	Fat, green
18	Aldehydes	Hexanal	5.83	1026/1051	Fat, grass, tallow
19		2‐(E)‐Heptenal	16.3	1257/1306	Fat, soap, almond
20		Nonanal	20.6	1328/1354	Fat, green, citrus
21		2‐(E)‐Octenal	22.45	1359/1392	Fat,green, nut,
22		2‐(E)‐Nonenal	28.84	1464/1509	Fat, green,cucumber
23		2‐(E)‐Decenal	35.07	1575/1597	Tallow
24	Others	2‐Pentylfuran	11.65	1171/1215	Green bean, butter
25		Phenol	46.44	1932/1965	Phenol

^a^ Retention index calculated based on published literature (Van Den Dool & Kratz, 1963).

^b^ Retention index of compounds in NIST 14 library.

**Table 3:** Identification and quantification of volatile components in bread using SPME‐ GC‐MS

Grp.	No.	Volatile	RI^a^/RI^b^	Control^c^	ARs‐B^d^	Sign.^e^
				(μg/kg)	(μg/kg)	
Alcohols	1	Ethanol	913/944	2,483 ± 322	2,365 ± 598	ns
	2	2‐Methyl‐1‐propanol	1044/1086	158 ± 32	132 ± 39	ns
	3	3‐Methyl‐1‐butanol	1166/1185	274 ± 95	620 ± 154	*
	4	1‐Pentanol	1200/1232	6 ± 2	9 ± 3	ns
	5	1‐Hexanol	1288/1336	33 ± 11	48 ± 9	*
	6	1‐Octen‐3‐ol	1380/1399	8 ± 2	8 ± 2	ns
	7	2‐Ethyl‐1‐hexanol	1426/1475	11 ± 4	11 ± 2	ns
	8	2‐Furanmethanol	1582/1620	95 ± 3	105 ± 5	ns
	9	2‐Phenylethanol	1829/1875	309 ± 113	1,020 ± 282	*
	10	2‐Methoxy‐4 vinylphenol	2117/2151	32 ± 12	40 ± 8	ns
Total content of alcohols				3,277 ± 166	4,067 ± 396	*
Esters	11	Ethyl octanoate	1373/1412	20 ± 1	13 ± 1	*
	12	γ‐Nonalactone	1961/2011	80 ± 15	87 ± 10	ns
	13	Ethyl myristate	2021/2044	7 ± 3	11 ± 1	ns
Total content of esters				109 ± 10	111 ± 8	ns
Aldehydes	14	2‐Methylbutanal	901/937	4 ± 1	7 ± 1	**
	15	Hexanal	1019/1051	28 ± 11	29 ± 9	ns
	16	Nonanal	1327/1354	19 ± 7	26 ± 6	ns
	17	2‐(E)‐Octenal	1358/1392	2 ± 1	9 ± 2	*
	18	Furfural	1386/1432	24 ± 6	28 ± 5	ns
	19	Benzaldehyde	1439/1478	151 ± 34	169 ± 53	ns
	20	2‐(Z)‐Nonenal	1464/1478	51 ± 1	64 ± 12	ns
	21	2,4‐(E,E)‐decadienal	1740/1789	89 ± 1	82 ± 18	ns
Total content of aldehydes				377 ± 43	431 ± 88	ns
Acids	22	Acetic acid	1376/1424	122 ± 49	162 ± 33	ns
	23	2‐Methylpropanoic acid	1489/1520	16 ± 1	18 ± 9	ns
	24	2‐Methylbutanoic acid	1589/1620	34 ± 4	52 ± 10	*
	25	Hexanoic acid	1766/1802	77 ± 17	98 ± 11	*
	26	Octanoic acid	1991/2038	85 ± 10	123 ± 13	*
	27	Nonanoic acid	2102/2144	118 ± 18	102 ± 29	ns
Total content of acids				451 ± 82	552 ± 53	ns
Ketones	29	2,3‐Butanedione	943/955	18 ± 3	39 ± 5	**
	28	Acetoin	1211/1253	326 ± 50	1,100 ± 299	*
Total content of ketones				343 ± 51	1,200 ± 338	*
Heterocycles	30	2‐Pentylfurane	1169/1215	8 ± 2	36 ± 5	ns
	31	2,5‐Dimethylpyrazine	1255/1300	8 ± 2	12 ± 3	ns
	32	2‐Acetylfuran	1425/1461	6 ± 2	13 ± 2	ns
	33	Maltol	1889/1938	ND	37 ± 12	*
Total content of heterocycles				23 ± 1	96 ± 13	**

^a^ Retention index calculated based on published literature (Van Den Dool & Kratz, 1963).

^b^ The published retention index of compounds in NIST 14 library.

^c^ Control: Normal bread; ND: non detected.

^d^ ARs‐B: Bread supplemented with ARs (0.3% w/w).

^e^ * and ** indicates significance at *p* < .05 and *p* < .01, respectively, and ns indicates no significant difference.

### 3.4 Quantitation of volatile compounds and calculation of odor activity values

Among the 33 identified and quantitated compounds, alcohols accounted for the highest proportion of volatile components both in control (3.24 mg/kg) and ARs‐B (4.06 mg/kg). ARs addition significantly improved the total content of alcohols, ketones and heterocycles by 25.6%, 250%, and 317%, respectively.

The concentrations of 3‐methyl‐1‐butanol (3, *malty‐like*), 1‐hexanol (5, *flowery‐like*), 2‐phenylethanol (9, *flowery‐like*), 2‐methylbutanal (14, *malty‐like*), 2‐(E)‐octenal (17, *fatty‐like*), 2‐methylbutanoic acid (24, s*weaty‐like*), hexanoic acid (25, *fatty‐like*), octanoic acid (26, *fatty‐like*), acetoin (28, *buttery‐like*), 2,3‐butanedione (29, *buttery‐like*) and maltol (33, *warmy‐fruity‐like*) were significantly increased in ARs‐supplemented bread compared with control (Table 3). Among these volatile compounds, the buttery‐like smelling acetoin had the highest concentration (1,100 μg/kg), followed by 2‐phenylethanol (1,020 μg/kg) and 3‐methyl‐1‐butanol (620 μg/kg). Analysis of odor activity values (OAVs) is an effective method for the verification of the volatile‐active compounds, because its evaluation criterion has taken into account the amounts of the compound and the odor threshold value (Birch et al., 2014). Therefore, in our study, OAVs of all compounds were calculated as the ratio of the volatile component concentration to odor threshold value determined in water. When OAV > 1, the volatile component was considered to contribute aroma to the sample. A total of 13 volatile components with OAV > 1 were listed in Table 4. Based on the volatile compounds with obvious difference in concentration between control and ARs‐B and the results of OAV, acetoin, 2,3‐butanedione, 3‐methyl‐1‐butanol, 2‐phenylethanol and 2‐methylbutanal were the main volatile compounds to the aroma of bread (Table 4). The OAVs of other aroma compounds such as 1‐hexanol, 2‐(E)‐octenal, 2‐methylbutanoic acid, hexanoic acid, octanoic acid and maltol were <1, suggesting these odorants may have a minor contribution to the overall aroma.

**Table 4:** Odor activity values of volatile compounds reaching a concentration above the odor threshold (OAV > 1)

Volatile compounds	Odor	Odor threshold (μg/kg)	Reference	OAV	
				Control^a^	ARs‐B^b^
2‐(Z)‐Nonenal	Fatty, tallowy, green	0.02	1	2,203	2,771
2,4‐(E,E)‐Decadienal	Deep fat fried, waxy	0.2	2	456	345
3‐Methyl‐1‐butanol	Balsamic, alcoholic, malty	4	1	99	185
Acetoin	Butterscotch, butter, yogurt, cream	14	3	26	56
Nonanal	Citrus, soapy	1.1	2	15	28
γ‐Nonalactone	Coconut‐like, sweet, fruity	9.7	4	9	10
1‐Octen‐3‐ol	Mushroom‐like	1.5	2	5	6
2‐Phenylethanol	Flowery, yeast‐like, honey	60	5	4	11
2‐Methoxy‐4 vinylphenol	Spicy	12.02	2	4	3
2,3‐Butanedione	Buttery, caramel	6	6	4	6
Hexanal	Green, grassy, tallow	5	2	3	4
2‐Pentylfuran	Butter, green bean, floral, fruity, mushroom, raw nuts	14.5	7	3	2
2‐Methylbutanal	Almond, malty	1	2	2	7

Note

[1]: Kirchhoff and Schieberle (2001); [2]: Giri et al. (2010); [3]: Boonbumrung et al. (2001); [4]: Czerny et al. (2008); [5]: Rashash et al., (1997); [6]: Leksrisompong et al. (2010); [7]: Aoki and Koizumi (1986).

^a^ Control: Normal bread.

^b^ ARs‐B: Bread supplemented with ARs (0.3% w/w).

Methyl‐1‐butanol and 2‐phenylethanol formed by decarboxylation reactions through Ehrlich pathway during dough fermentation might contribute to the malty and flowery characteristic aromas of wheat bread (Pico et al., 2015; Sahin & Schieberle, 2019). In our study, the concentration of 3‐methyl‐1‐butanol and 2‐phenylethanol in ARs‐B group was 620 μg/kg and 1,020 μg/kg, which was nearly 2.3 and 3.1 times higher than that in control bread. The result may explain the effects from quantitative descriptive analysis that ARs addition can improve the malty and flowery aroma of bread in some extent (Figure 2). Moreover, the addition of ARs significantly increased the concentration of acetoin and 2,3‐butanedione, which were the metabolites derived from the fermentation process of yeast and directly linked to the yeast activity, hinting that ARs might promote yeast fermentation activity and increase fermentation productions (Birch et al., 2014; Pico et al., 2015). Acetoin and 2,3‐butanedione were positively correlated with the characteristic aroma (buttery) in wheat bread and could be used as food flavoring and fragrance in bakery products due to their pleasant odor characterization (Birch et al., 2013; Di Renzo et al., 2018; Pico et al., 2015). In our present study, ARs addition significantly increased the content of acetoin and 2,3‐butanedione by 3.37 and 2.17 times compared with control bread, which could partially explain the result of sensory characterization that ARs‐supplemented bread pronounced predominantly buttery aroma compared with control bread. In addition, the concentration of 2‐methylbutanal in ARs‐B (7 μg/kg) was 1.75 times higher than that in control bread. 2‐Methylbutanal was also produced by the Ehrlich pathway (Martínez‐Anaya, 1996), which contributed a pleasant malt aroma in bread and could be responsible for the bread crust aroma because of its high OAV (Zehentbauer & Grosch, 1998). Taken together, the increased concentration of acetoin, 2,3‐butanedione, 3‐methyl‐1‐butanol, 2‐phenylethanol and 2‐methylbutanal, induced by ARs addition might be responsible for the flavor promotion of bread.

### 3.5 Effect of ARs on the texture of wheat bread

**Figure 4:**
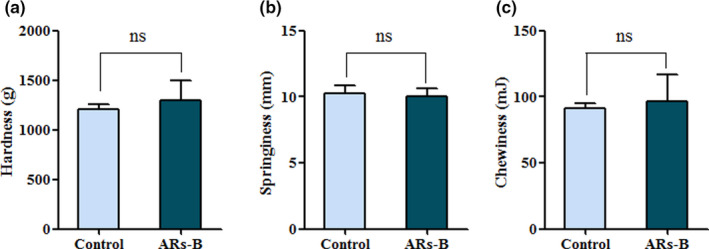
The effect of ARs addition on hardness, springiness, and chewiness of bread. (a) Hardness, (b) springiness, and (c) chewiness of bread were detected by texture analysis. Significant differences compared with control bread group are assessed by Student's *t* test. Level of significance: * indicates significance at *p* < .05. Control: Normal bread; ARs‐B: Bread supplemented with ARs (0.3% w/w)

As shown in Figure 4, there was no significant difference on hardness, springiness, and chewiness between control bread and ARs‐B, suggesting that ARs addition (3 g/kg) would not cause negative effect on bread texture. The results were consistent with the conclusion of sensory evaluation (Figure 3). However, a study from Annica *et al* found that ARs with a higher amount (5–10 g/kg) had a negative effect on bread texture, causing lower bread volume and baker's yeast activity (Annica Am et al., 2011). Therefore, ARs addition in this study not only enhanced the flavor profile of wheat bread, but also improved their sensory property and quality, possibly due to its lower supplementation or different homologues composition.

## 4 CONCLUSION

In our present study, the addition of ARs could significantly inhibit the formation of CML in wheat bread without affecting the texture properties of bread. Sensory evaluation result showed that ARs supplementation obtained a higher score in consumer overall acceptability and buttery‐like aroma characteristics compared with the control. Aroma compounds such as acetoin, 2,3‐butanedione, 3‐methyl‐1‐butanol, 2‐phenylethanol, and 2‐methylbutanal were identified as the main contributors to the bread flavor by ARs. The results indicated that ARs can be served as a novel functional food additive to improve the quality and flavor of baking products.

## CONFLICTS OF INTEREST

The authors declare that there are no conflicts of interest.

## ETHICAL GUIDELINES

Ethics approval was not required for this research.

## INFORMED CONSENT

Written informed consent was obtained from all study participants.

## Data Availability

Data available on request from the authors.
